# Thermodynamic Analysis of Methylcyclohexane Dehydrogenation and Solar Energy Storage via Solar-Driven Hydrogen Permeation Membrane Reactor

**DOI:** 10.3390/membranes10120374

**Published:** 2020-11-27

**Authors:** Hongsheng Wang, Bingzheng Wang, Hui Kong, Xiaofei Lu, Xuejiao Hu

**Affiliations:** 1 MOE Key Laboratory of Hydrodynamic Transients, School of Power and Mechanical Engineering, Wuhan University, Wuhan 430072, China; xjhu@whu.edu.cn; 2 Department of Chemical System Engineering, School of Engineering, The University of Tokyo, 7-3-1 Hongo, Bunkyo-ku, Tokyo 113-8656, Japan; xiaofei_l@chemsys.t.u-tokyo.ac.jp; 3 Department of Energy Engineering, Zhejiang University, Hangzhou 310027, China; wang_bz@zju.edu.cn; 4 Beijing Institute of Technology, School of Mechanical Engineering, Beijing 100081, China; konghui2020@bit.edu.cn; 5 Hubei International Scientific and Technological Cooperation Base of Sustainable Resource and Energy, Wuhan University, Wuhan 430079, China

**Keywords:** MCH dehydrogenation, thermodynamic analysis, hydrogen generation, membrane reactor, solar thermochemistry

## Abstract

A novel methylcyclohexane (MCH) dehydrogenation system driven by solar energy with a hydrogen permeation membrane (HPM) reactor is proposed in this study. It is a promising method, via this novel system, to generate pure hydrogen and store intermittent solar energy. In this research, the thermodynamic analysis of MCH dehydrogenation via the HPM reactor was conducted based on numerical simulation. The conversion rates and thermodynamic efficiencies under different temperatures (150–350 °C), permeate pressures from 0.001 to 0.5 bar, and solar irradiation in the four seasons were studied and analyzed. Under a hydrogen partial pressure difference, HPM can separate hydrogen and shift the reaction equilibrium forward for a higher conversion rate of MCH, which can reach nearly 99.7% in this system. The first-law of thermodynamic efficiency, the solar-to-fuel efficiency, and the exergy efficiency are up to 95.58%, 38.65%, and 94.22%, respectively. This study exhibits the feasibility and potential of MCH dehydrogenation via the HPM reactor driven by solar energy and provides a novel approach for solar energy storage.

## 1. Introduction

Considered as a green energy carrier, hydrogen is becoming a viable clean choice of energy storage and transportation, and it can be further utilized for power generation by fuel cells [1,2,3]. Hydrogen is widely used for hydrocracking and desulphurization purposes, fertilizer production, food processing, etc. [4]. However, compared to fossil fuels, gaseous hydrogen has a low energy density by volume, and a safe and economical hydrogen storage system is crucial and necessary for hydrogen utilization [5]. In the past few decades, many kinds of hydrogen storage systems have been studied, such as physical-based hydrogen storage (e.g., compressed gas, liquid hydrogen), chemical sorption hydrogen storage (e.g., metal hydrides, organic hydrides), physical sorption (e.g., carbon materials, metal-organic framework) and organic hydride hydrogen storage methods [4]. The organic hydride hydrogen storage method is attractive due to the carbon-free store and release processes. Moreover, the organic carrier can be used repeatedly, and the storage pressure is low [6]. There are two main organic hydride hydrogen storage systems based on the dehydrogenation reaction: the cyclohexane (CH)–benzene (BZ) system and the methylcyclohexane (MCH)–methylbenzene (TOL) system. MCH and TOL in the MCH dehydrogenation system have lower melting points (−126 °C for MCH, −95 °C for TOL) than the compounds in the CH–BZ system (6.5 °C for CH, 5.5 °C for BZ), which means that MCH can be stably stored as a liquid at room temperature [7,8,9]. The hydrogen release process of MCH dehydrogenation can be expressed as:

(1) $$C_{7}H_{14}\left(g\right)⇌C_{7}H_{8}\left(g\right){+3H}_{2}\left(g\right),\;\Delta H_{25\;{}^{\circ}C}^{{}^{⊖}}=204.8\;\mathrm{kJ}/\mathrm{mol}$$

However, the MCH dehydrogenation process is thermodynamically constrained, especially at low temperatures, where the conversion rate is low [10]. Many scholars used a hydrogen permeation membrane (HPM) to improve the conversion rate of the MCH dehydrogenation process: Jawad et al. used a palladium (Pd)–silver (Ag) membrane to separate hydrogen, and the conversion rate was up to four times higher than the equilibrium value after 300 h onstream and repeated temperature cycling [11]. Meng et al. improved the MCH dehydrogenation conversion rate with a hydrogen-selective organosilica membrane reactor; the improvement was from 44% to 86% at 250 °C, 101.3 kPa with a permeate pressure of 10 kPa [12]. Cholewa et al. simulated MCH dehydrogenation based on a Pd membrane reactor, and they achieved an MCH conversion rate of 90% and hydrogen recovery above 80% under the conditions: 350 °C, 30 bar with a permeate pressure of 3 bar [13]. Many kinds of HPM materials have the potential to be used in this novel system, such as a polymeric membrane, a perovskite membrane, a ceramic membrane, and a metallic membrane. It was observed that for many hydrogen-selective membranes, the Pd–Ag membrane can greatly release the hydrogen embrittlement [14,15] and has relatively high selectivity and H_2_ flux [16]; the Pd–Ag membrane was chosen as the HPM in this research. A common catalyst of MCH dehydrogenation is Pt/γ–Al_2_O_3_, due to its good activity [17,18,19], which is utilized in this kinetic and thermodynamic simulation.

For the traditional MCH dehydrogenation reactor, the consumed thermal energy for driving this endothermic reaction is usually supplied by fossil fuel or electricity, which will lead to greenhouse gas emission (e.g. CO_2_) directly or indirectly [20]. Using clean, abundant, and widespread solar energy as a heat source to power MCH dehydrogenation is environmentally friendly. In the past few years, concentrated solar energy (CSE), which can reduce the cost of fossil fuel and the emission of carbon dioxide, has developed rapidly and researches on solar thermochemical reactions attracted much attention. Kong et al. proposed a strategy for H_2_O/CO_2_ splitting for H_2_/CO generation with enhanced efficiency (24.36% without heat recovery) via a thermochemical cycle system driven by CSE [21]. Wang et al. studied a membrane reactor with a parabolic trough collector for ammonia decomposition in mid-low temperature [22]. Hong et al. theoretically and experimentally studied the integration between a solar trough collector and a methanol reforming reaction or methanol decomposition reaction at around 200–400 °C [23]. For most solar thermochemical reactions, solar collectors are used for collecting solar energy. There are three main solar collectors: the solar trough collector, the solar dish collector, and the solar tower collector. The concentrating method of the dish collector and the tower collector is point focusing, which is usually used for the mid/high-temperature range [24,25,26]. For the temperature range in this paper (150–350 °C), the line-focused solar trough collector, whose heating temperature is below 500 °C [24,25], is chosen to collect solar energy due to its high commercialization and low cost [22,27]. The relatively low heating temperature of the trough collector causes relatively small heat loss, leading to a high absorbing efficiency. In the process of MCH dehydrogenation heated by a solar trough collector, the mid/low-temperature solar energy with low energy level is converted into chemical energy, which has a relatively high energy level, and there is an improvement in the energy level, which is defined as the ratio of exergy change to enthalpy change during the energy release process [28].

In the existing literature, the MCH dehydrogenation process has been widely studied, while the mainly focused has been on the kinetics and catalysis process; the thermodynamic and environmental performance of MCH dehydrogenation via HPM driven by solar energy, which is also crucial for further experiment and industrial application, has not been researched. Therefore, in this research, a novel solar-driven MCH dehydrogenation system integrated with an HPM reactor was first proposed and analyzed in thermodynamics and kinetics. The conversion rate, first–law of thermodynamic efficiency, solar–to–fuel efficiency, and exergy efficiency were researched and optimized. The system environment performance was also gauged by the standard coal saving rate (SCSR) and carbon dioxide emission reduction rate (CDRR).

## 2. System Description

**Figure 1:**
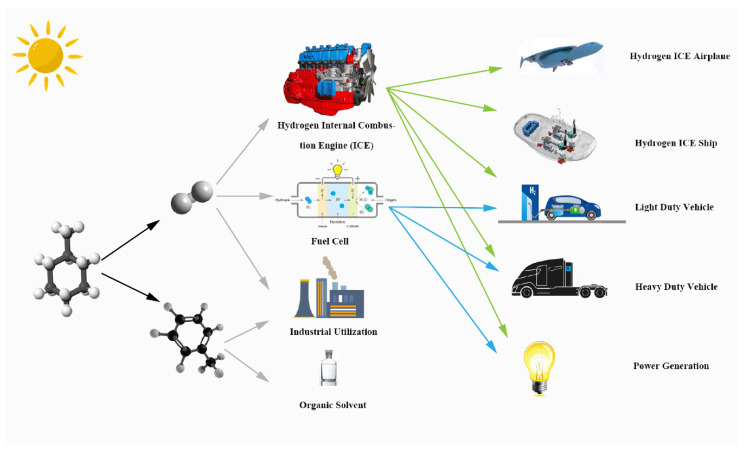
Graphical illustration of solar-driven MCH dehydrogenation system.

Figure 1 illustrates the products of this solar-driven MCH dehydrogenation system and their further utilization. MCH is a common hydrogen storage organic; the products of MCH dehydrogenation reaction are hydrogen and TOL. The high purity hydrogen of this system can supply the fuel cell and hydrogen energy automobile. Hydrogen and TOL can be used as raw materials for industrial production, and TOL is a common organic solvent. 

**Figure 2:**
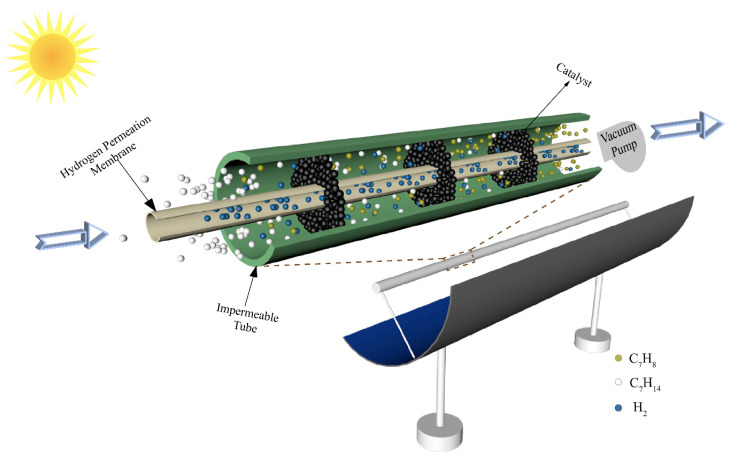
Schematic of a conceptual solar-driven MCH dehydrogenation membrane reactor equipment.

The schematic of a conceptual solar-driven MCH dehydrogenation membrane reactor equipment is shown in Figure 2. The solar parabolic trough collector collects the solar thermal energy by focusing sunlight on the reactor located in the focal line of the collector. The reactor consists of an impermeable tube, an HPM deposited on a porous alumina support, and catalysts. The Pd–Ag membrane is chosen as the HPM in this paper due to its high selectivity and high hydrogen flux compared with other kinds of hydrogen permeation membrane. The Pd–Ag membrane used in this paper is prepared by the deposition of atomic layers on porous ceramic substrates. The interior tube is HPM and a vacuum pump is located in the chamber of the HPM (separation side) to maintain a negative permeate pressure for hydrogen separation. The exterior tube is impermeable and the chamber between the interior tube and exterior tube (feed side) is filled with 1.0 wt% Pt/γ–Al_2_O_3_ catalyst. The inner radius (R_in_) and outer radius (R_out_) of the HPM tube are 0.95 and 1 cm, respectively. When the system is working, the MCH is preheated to reaction temperature by solar thermal energy and then flows into the reactor at a constant rate (30 sccm, standard milliliter per minute, in this research). In the process of reaction, the hydrogen permeates through the membrane under the pressure difference between the feed side and the separation side, which increases the final conversion rate. It must be emphasized that the thermodynamic analysis results of this system are under the following assumptions [28]: (a) the gas diffusion from downstream to upstream of the tube is neglected; (b) all gases are ideal gases that conform to the ideal state equation; (c) the flow resistance is negligible; (d) all potential side products and reactions were ignored; (e) the HPM is assumed to separate H_2_ and block other gases; and (f) the pressure drop along the tube is not taken into account. 

**Figure 3:**
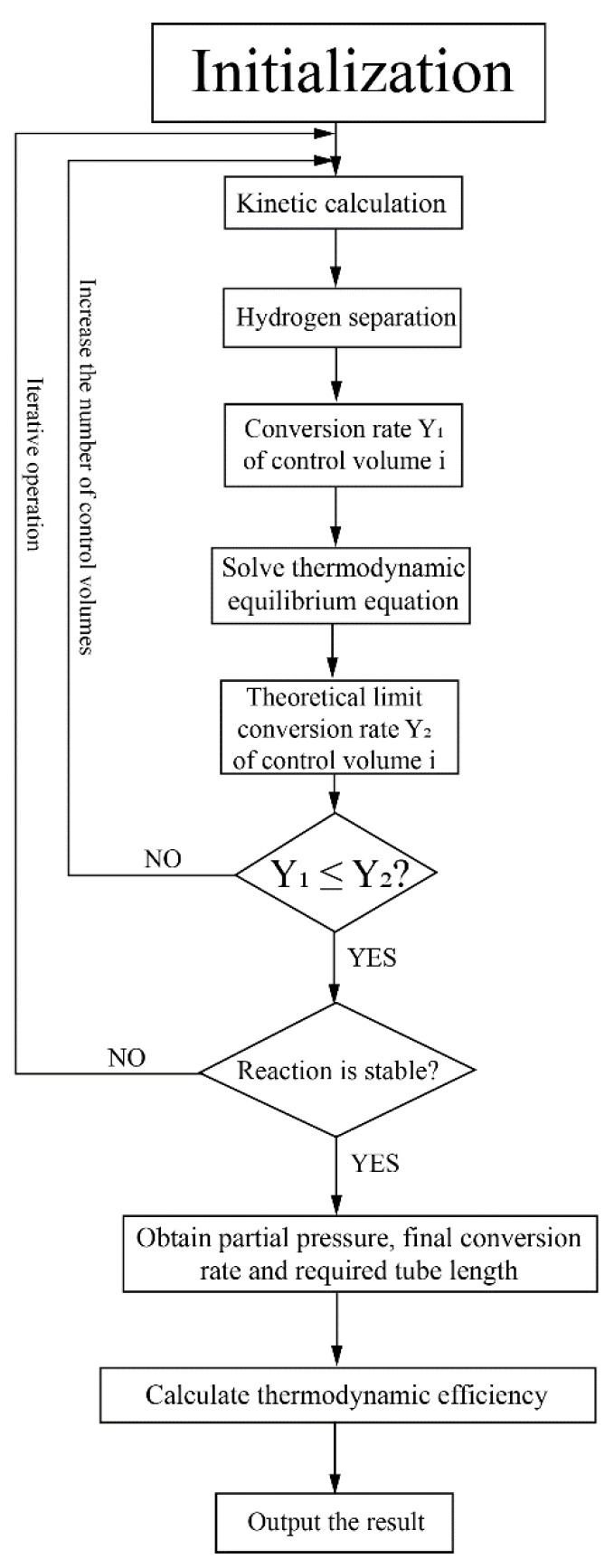
Program flow chart for the simulation of the solar-driven MCH dehydrogenation system.

This system was studied based on numerical simulation and the program flow chart for this system is shown in Figure 3. The HPM reactor was divided into several control volumes along the axial direction for simulation. Based on initial gas partial pressures in the reactor, the kinetic calculation was conducted and the conversion rates in each control volume were obtained. The theoretical limit conversion rate was also calculated according to the thermodynamic equilibrium equation to verify the rationality of the kinetic calculation. The hydrogen separation flux and amount were calculated based on the hydrogen separation model. After the reaction reached a stable state (the relative variation of conversion rate between two adjacent control volumes is less than 0.1% in this research), the final conversion rate and the required tube length were obtained, which can be used to calculate the thermodynamic efficiency. The kinetic model of MCH, the hydrogen separation model and the method to calculate thermodynamic efficiency will be discussed in the next section and Appendix A.

## 3. Theoretical Formulation

The first–law of thermodynamic efficiency, which reflects the conversion efficiency of the energy amount in the process of MCH dehydrogenation, can be defined as the ratio of energy output (hydrogen and TOL chemical energy) to the energy input (solar energy and MCH chemical energy), expressed as [29]:

(2) $$\eta_{\mathrm{HHV}}=\frac{n_{H_{2}}\cdot {\mathrm{HHV}}_{H_{2}}+n_{C_{7}H_{8}}\cdot {\mathrm{HHV}}_{C_{7}H_{8}}}{\eta_{\mathrm{opt}}^{-1}\eta_{\mathrm{abs}}^{-1}\cdot (Q_{\mathrm{preheat}}+Q_{\mathrm{enthalpy}})+\eta_{s\to e}^{-1}\cdot (W_{p,\mathrm{vacuum}}+W_{p,\mathrm{compressor}})+n_{C_{7}H_{14}}\cdot {\mathrm{HHV}}_{C_{7}H_{14}}}$$

where $n_{C_{7}H_{14}}$ is the consumed molar amount of MCH; $n_{H_{2}}$ and $n_{C_{7}H_{8}}$ are the molar amounts of hydrogen and TOL generated in the system, respectively; ${\mathrm{HHV}}_{H_{2}}$, ${\mathrm{HHV}}_{C_{7}H_{8}}$, and ${\mathrm{HHV}}_{C_{7}H_{14}}$ are the molar higher heating values of hydrogen, TOL, and MCH, taken as 285.8, 3947.85, and 4600.58 kJ mol^−1^ [30], respectively; *η*_abs_ is the absorption efficiency, which is assumed as 90% in this research [28,31]; *η*_opt_ is the optical efficiency of parabolic trough collector, taken as 73% [28]; *η*_s__→e_ is solar-to-electricity efficiency, taken as 15% for commercial photovoltaic (PV) cells and 40% for state-of-the-art PV efficiency in the laboratory. *Q*_preheat_ is the solar thermal energy consumed for raising the MCH temperature from room temperature to reaction temperature. *Q*_enthalpy_ is the solar thermal energy consumed for the enthalpy change of MCH dehydrogenation. *W*_p,vacuum_ is the exergy consumed by a vacuum pump for hydrogen separation. *W*_p,compressor_ is the exergy consumed by a compressor for feeding the MCH into the reactor. The calculation equations of the above energy items are listed in Appendix A. It must be emphasized that *W*_p,vacuum_ and *W*_p,compressor_ are the exergies (minimum energy) consumed, which are used to calculate the upper bound of thermodynamic efficiency and exhibit the potential of this system for further utilization. The vacuum pump efficiency is defined as the ratio of required exergy to the practical consumed energy of the vacuum pump, which can be expressed as [32,33]:

(3) $$\eta_{p_{1}}={\left(\frac{P_{H_{2,\mathrm{out}}}}{P^{⊖}}\right)}^{0.544}$$

where $\eta_{p_{1}}$ is the vacuum pump efficiency and $P^{⊖}$ is the standard pressure. The compressor mechanical efficiency $\eta_{p_{2}}$ is taken as 85% in this research [34]. After taking vacuum pump efficiency and compressor mechanical efficiency into consideration, the first–law of thermodynamic efficiency is defined as:

(4) $$\eta_{\mathrm{HHV},\mathrm{real}}=\frac{n_{H_{2}}\cdot {\mathrm{HHV}}_{H_{2}}+n_{C_{7}H_{8}}\cdot {\mathrm{HHV}}_{C_{7}H_{8}}}{\eta_{\mathrm{opt}}^{-1}\eta_{\mathrm{abs}}^{-1}\cdot (Q_{\mathrm{preheat}}+Q_{\mathrm{enthalpy}})+\eta_{s\to e}^{-1}\cdot (\eta_{p_{1}}^{-1}\cdot W_{p,\mathrm{vacuum}}+\eta_{p_{2}}^{-1}\cdot W_{p,\mathrm{compressor}})+n_{C_{7}H_{14}}\cdot {\mathrm{HHV}}_{C_{7}H_{14}}}$$

The first–law of thermodynamic efficiency expressed by Equations (2) and (4) shows the energy utilization ability of this system, while the chemical energy of products comes from both solar energy and MCH chemical energy. To measure the system capability of converting solar energy into chemical energy, the solar-to-fuel efficiency which eliminates the contribution of MCH chemical energy to convert into chemical energy can be defined as the ratio of chemical energy increment to solar energy input [35], expressed as:

(5) $$\eta_{s\to f}=\frac{n_{H_{2}}\cdot {\mathrm{HHV}}_{H_{2}}+n_{C_{7}H_{8}}\cdot {\mathrm{HHV}}_{C_{7}H_{8}}-n_{C_{7}H_{14}}\cdot {\mathrm{HHV}}_{C_{7}H_{14}}}{\eta_{\mathrm{opt}}^{-1}\eta_{\mathrm{abs}}^{-1}\cdot (Q_{\mathrm{preheat}}+Q_{\mathrm{enthalpy}})+\eta_{s\to e}^{-1}\cdot (W_{p,\mathrm{vacuum}}+W_{p,\mathrm{compressor}})}$$

(6) $$\eta_{s\to f,\mathrm{real}}=\frac{n_{H_{2}}\cdot {\mathrm{HHV}}_{H_{2}}+n_{C_{7}H_{8}}\cdot {\mathrm{HHV}}_{C_{7}H_{8}}-n_{C_{7}H_{14}}\cdot {\mathrm{HHV}}_{C_{7}H_{14}}}{\eta_{\mathrm{opt}}^{-1}\eta_{\mathrm{abs}}^{-1}\cdot (Q_{\mathrm{preheat}}+Q_{\mathrm{enthalpy}})+\eta_{s\to e}^{-1}\cdot (\eta_{p_{1}}^{-1}\cdot W_{p,\mathrm{vacuum}}+\eta_{p_{2}}^{-1}\cdot W_{p,\mathrm{compressor}})}$$

where Equations (5) and (6) are the solar-to-fuel efficiencies with pump exergy and with real pump energy.

The solar-to-fuel efficiency and the first-law thermodynamic efficiency mainly focus on the conversion of the energy amount, while the conversion of energy quality in this research is also significant, which can be defined as the ratio of output exergy to the input exergy of this system [22,28,36], given as:

(7) $$\eta_{\mathrm{ex}}=\frac{n_{C_{7}H_{8}}\cdot Ex_{C_{7}H_{8}}+n_{H_{2}}\cdot Ex_{H_{2}}+Q_{\mathrm{wh}}\cdot (1-\frac{T_{0}}{T_{H}})}{Ex_{\mathrm{solar}}+n_{C_{7}H_{14}}\cdot Ex_{C_{7}H_{14}}+W_{p,\mathrm{vacuum}}+W_{p,\mathrm{compressor}}}$$

(8) $$Ex_{\mathrm{solar}}=\left(1-\frac{4T_{0}}{3T_{\mathrm{sun}}}+\frac{1}{3}\cdot {\left(\frac{T_{0}}{T_{\mathrm{sun}}}\right)}^{4}\right)\cdot \eta_{\mathrm{opt}}^{-1}\eta_{\mathrm{abs}}^{-1}\cdot (Q_{\mathrm{preheat}}+Q_{\mathrm{enthalpy}})$$

where $Ex_{\mathrm{solar}}$ is the input of solar exergy; $T_{\mathrm{sun}}$ is the surface temperature of the sun, taken as 5800 K; $Ex_{C_{7}H_{8}}$, $Ex_{H_{2}}$, and $Ex_{C_{7}H_{14}}$ are the chemical exergy of TOL, hydrogen, and MCH, given as 3928.3, 235.2, and 4556.9 kJ/mol, respectively [30]; and *Q*_wh_ is the thermal energy contained in the products after the reaction.

### Environmental Performance Calculation

The required thermal energy of this system comes from solar energy, which can save fossil fuels without producing carbon emission. Thus, the standard coal saving rate (SCSR) and carbon dioxide emission reduction rate (CDRR) are calculated to measure the environmental performance of this system. Assuming that the absorbed solar energy and energy consumed by vacuum pump and compressor are provided by standard coal, the SCSR and CDRR can be defined as:

(9) $$SCSR=\frac{\eta_{c\to h}^{-1}({\dot{Q}}_{\mathrm{preheat}}^{}+{\dot{Q}}_{\mathrm{enthalpy}})+\eta_{c\to e}^{-1}\cdot (\eta_{p_{1}}^{-1}\cdot {\dot{W}}_{p,\mathrm{vacuum}}^{}+\eta_{p_{2}}^{-1}\cdot {\dot{W}}_{p,\mathrm{compressor}}^{})}{q_{\mathrm{coal}}}$$

(10) CDRR=SCSR⋅μ

where ${\dot{Q}}_{\mathrm{preheat}}^{}$, ${\dot{Q}}_{\mathrm{enthalpy}}^{}$, ${\dot{W}}_{p,\mathrm{vacuum}}^{}$, and ${\dot{W}}_{p,\mathrm{compressor}}^{}$ are preheat thermal energy, enthalpy energy, energy for separation and for feeding reactant consumed at per unit time and per unit mirror field area of the solar collector, respectively; *η*_c__→h_ and *η*_c__→e_ are the conversion efficiencies from standard coal to heat and to electricity, respectively, taken as 80% and 40%, respectively [37,38]; $q_{\mathrm{coal}}$ is the heating value of standard coal, which is 2.931 × 10^4^ kJ/kg; and *μ* is the mass ratio of carbon dioxide emission to standard coal combustion, taken as 2.45 [31].

## 4. Results and Discussion

This novel system was studied by numerical simulation under the temperatures from 150 to 350 °C and the permeate pressures in the range of 0.001 to 0.5 bar. In this section, the final conversion rate, energy efficiency, required tube length, SCSR, and CDRR are shown and discussed under different temperatures and permeate pressures. The energy efficiencies at different feeding pressures and different solar irradiation in a year were also investigated and optimized. The flow rate is 30 sccm and the feeding pressure is 1 bar unless stated otherwise.

### 4.1. Conversion Rate

**Figure 4:**
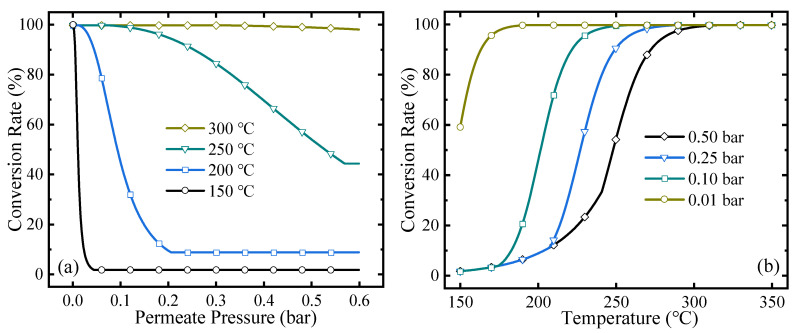
MCH conversion rates under (**a**) different permeate pressures and (**b**) reaction temperatures.

The conversion rate can directly reflect the extent of the reaction. The MCH dehydrogenation conversion rates under different permeate pressures and temperatures are shown in Figure 4. As the permeate pressure increases, the conversion rate decreases because higher permeate pressure corresponds to higher final hydrogen partial pressure in the reactor, which blocks further shift of the equilibrium of reaction and leads to a lower conversion rate at a certain temperature. For the variation of temperature, when the permeate pressure is equal to or higher than the hydrogen partial pressure in the equilibrium state, no hydrogen can be separated and the HPM reactor does not work. In Figure 4b, with the temperature decreasing, the conversion rate goes down as MCH dehydrogenation is an endothermic reaction, and the hydrogen partial pressure in the equilibrium state inside the HPM also increases. When it equals the permeate pressure, the HPM reactor does not work, and the conversion rate of MCH dehydrogenation equals that under equilibrium state at a certain temperature. In Figure 4, increasing temperature and decreasing permeate pressure can obtain a higher conversion rate, which can reach 99.7% under 300 °C, 0.5 bar or 200 °C, 0.01 bar, while higher temperature consumes more thermal energy and lower permeate pressure corresponds to a larger amount of separation energy consumption. Therefore, the energy efficiencies at different working conditions need to be analyzed and optimized.

### 4.2. Thermodynamic Efficiency Analyses

**Figure 5:**
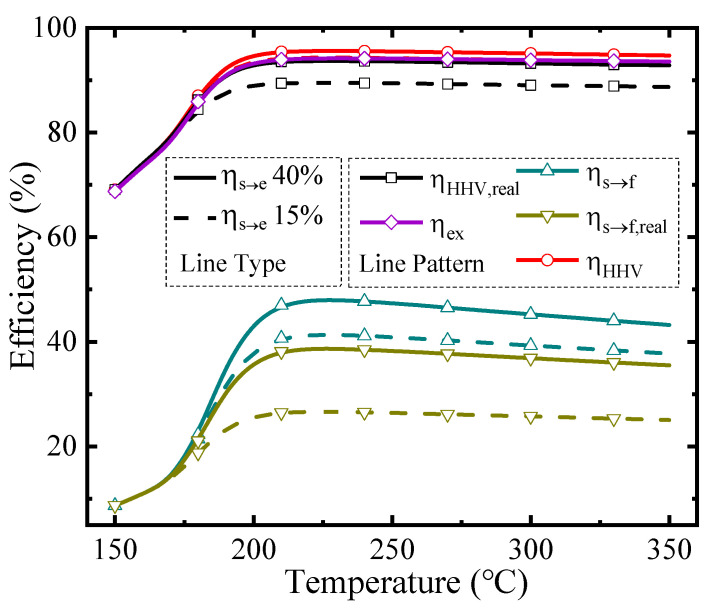
Thermodynamic efficiencies under the permeate pressure of 0.1 bar.

**Figure 6:**
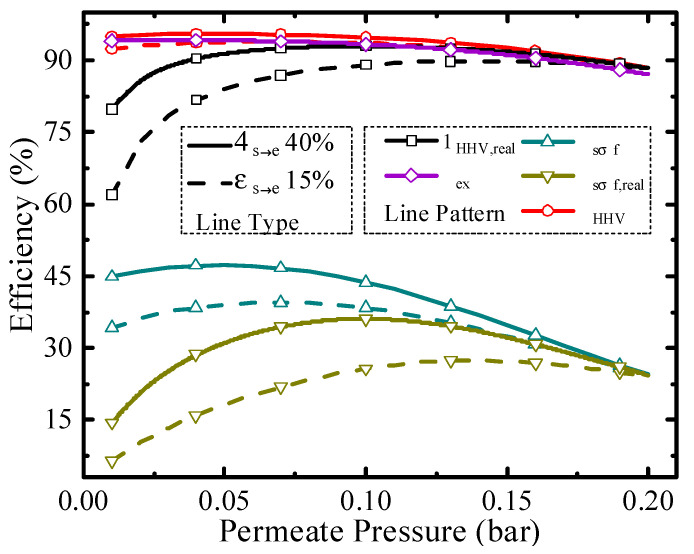
Thermodynamic efficiencies under different permeate pressures at 200 °C.

The thermodynamic efficiencies at different temperatures under a permeate pressure of 0.1 bar are shown in Figure 5. The line type represents different solar-to-electricity efficiency values utilized in the thermodynamic calculation (Equations (2) and (4)–(6)), and the line pattern distinguishes different kinds of thermodynamic efficiencies in this research, which is similar to Figure 6. The efficiencies with separation exergy (Equations (2) and (5)) indicate the upper bounds of efficiencies, which have the potential to be achieved by upgrading the separation method; and efficiencies with real separation energy (Equations (4) and (6)) correspond to the state-of-the-art efficiencies in the industry and can be achieved by the common separation method (e.g., vacuum pump separation). As the temperature rises, all the efficiencies increase initially and then go down. Two main factors influence these efficiencies. On the one hand, rising temperature increases the conversion rate, so more solar energy can be converted into chemical energy instead of dissipation as thermal energy, resulting in higher efficiencies. On the other hand, according to Equations (A9) and (A10), the thermal energy consumed for preheating reactants and the enthalpy change is larger at high temperatures, which decreases the efficiency. Under the influence of these two factors, which have the opposite effect on efficiencies, there are maximum efficiency values and optimal working conditions. With the *η*_s__→e_ of 40%, the first-law of thermodynamic efficiency, solar-to-fuel efficiency, and exergy efficiency can reach 95.58%, 38.65%, and 94.16% under 0.1 bar, and 230 °C, respectively.

**Figure 7:**
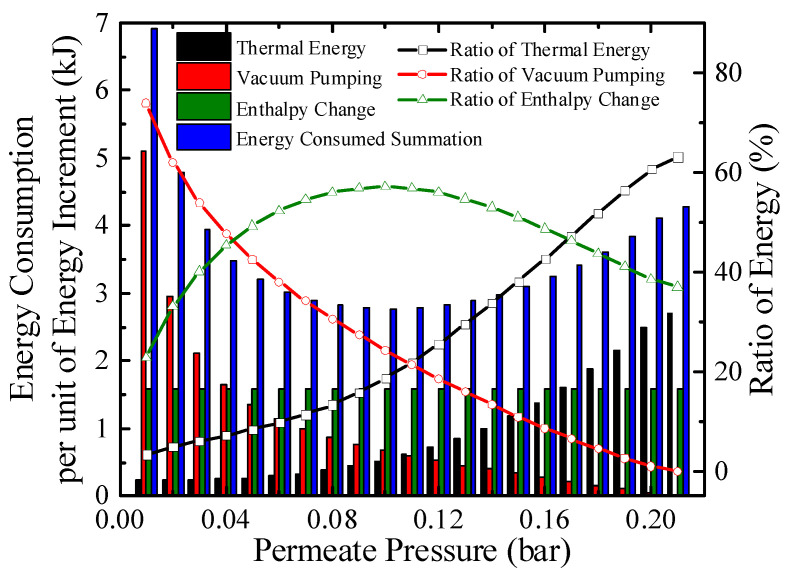
Energy consumption per unit energy increment with real separation energy at 200 °C (*η*_s__→e_ = 40%).

Permeate pressure is another significant parameter for the evaluation of thermodynamic performance, and it affects the conversion rate, pump efficiency, and separation exergy of H_2_. Thermodynamic efficiencies under different permeate pressures at 200 °C are shown in Figure 6. As the permeate pressure increases, the hydrogen partial pressure inside the HPM reactor also increases, leading to a lower conversion rate. The higher permeate pressure consumes less separation energy, which is beneficial to the increment of thermodynamic efficiency. Due to the influence of permeate pressure on thermodynamic efficiency with two opposite directions, the optimal working conditions exist, such as 0.04 bar for exergy efficiency (94.17%) with real separation energy under 40% *η*_s__→e_ and 0.14 bar for solar-to-fuel efficiency (27.40%) with real separation energy under 15% *η*_s__→e_. The above analyses are qualitative, and to analyze the efficiency change trend concretely, quantitative analysis was also conducted and given in Figure 7.

The energy consumption per unit energy increment with real separation energy at 200 °C is qualitatively exhibited in Figure 7. The consumption consists of thermal energy for reactants preheating and enthalpy change, and energy for separation hydrogen. At a certain temperature, the enthalpy change for per unit energy increment is a constant. The thermal energy consumption per unit energy increment increases as permeate pressure goes up because the energy for preheating is constant, a lower conversion rate at higher permeate pressure indicates less chemical energy obtained. For per unit energy increment, the amount of hydrogen generated is a constant, and according to Equations (3) and (A11), the separation energy for per unit hydrogen generation increases as permeate pressure goes down. Therefore, the energy consumed summation has a minimum value under 0.1 bar, 200 °C in Figure 7 which corresponds to the maximum efficiency, where the thermal energy, enthalpy change, separation energy consumption for per unit energy increment are 0.51, 1.58, and 0.67 kJ, respectively.

**Figure 8:**
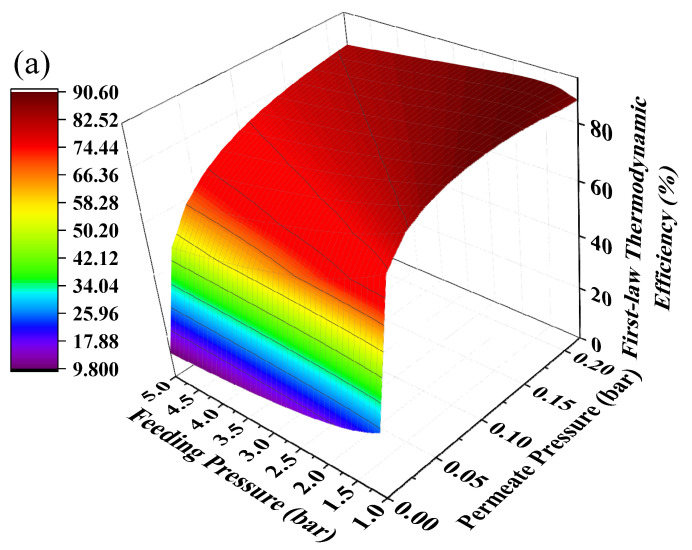

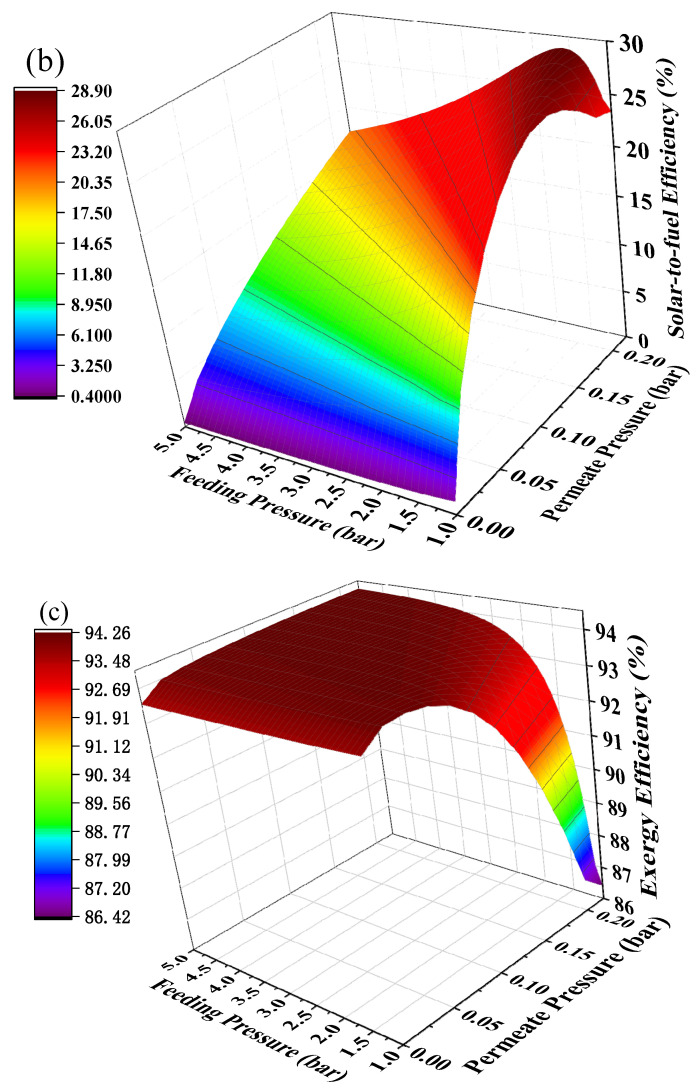
Thermodynamic efficiencies under different permeate pressures and feeding pressures at 200 °C with real separation energy (*η*_s__→e_ = 15%). (**a**) the first–law thermodynamic efficiency; (**b**) solar–to–fuel efficiency; (**c**) exergy efficiency.

Hydrogen separation is driven by pressure difference and using positive feeding pressure supported by a compressor to feed MCH into the HPM reactor can contribute to pressure difference between two sides of the membrane. The mechanical efficiency (~85%) of the compressor is much higher than vacuum pump efficiency, thus the thermodynamic efficiencies under the combination of positive feeding pressure and negative permeate pressure have the potential to be further improved. The thermodynamic efficiencies at different permeate pressures and feeding pressures under 200 °C with real separation energy are shown in Figure 8. The first-law of thermodynamic efficiency and solar-to-fuel efficiency with a compressor can reach as high as 90.51% and 28.88% under a feeding pressure of 1.7 bar and permeate pressure of 0.21 bar at 200 °C, and exergy efficiency reaches 94.22% under a feeding pressure of 2.2 bar and permeate pressure of 0.1 bar. Without the compressor, the first–law thermodynamic efficiency and solar–to–fuel efficiency can reach 89.86% and 27.40% under a permeate pressure of 0.14 bar, and the exergy efficiency is 94.17% under a permeate pressure of 0.04 bar, given in Figure 6. Therefore, a positive feeding pressure driven by a compressor can improve the thermodynamic performance of this system.

**Figure 9:**
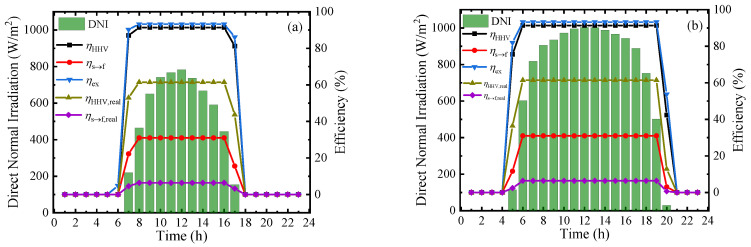

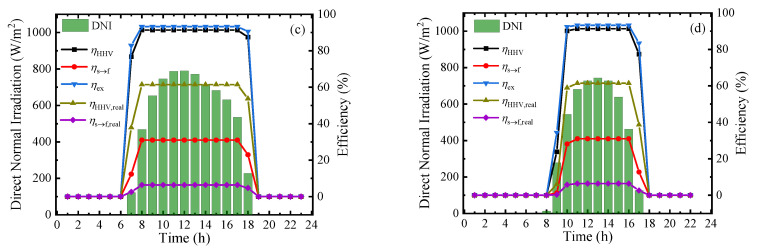
Variation of direct normal irradiation (DNI) and thermodynamic efficiencies on typical sunny days of different months under 350 °C, 0.01 bar (*η*_s__→e_ = 15%): (**a**) March; (**b**) June; (**c**) September; and (**d**) December.

To measure the performance of this system at a practical scene, the hourly direct normal irradiation (DNI) and corresponding thermodynamic efficiencies on typical sunny days in Beijing [39] in four seasons are shown in Figure 9. From the results, the thermodynamic efficiencies can be maintained at a stable and high level in the daytime, and the first–law thermodynamic efficiency, solar–to–fuel efficiency, and exergy efficiency can maintain at 91.34%, 30.99%, and 93.22%, respectively. However, due to the difference of irradiation time, the system working time at optimal efficiency is various in a year and daily working hours in June are almost twice as long as that in December. The result of Figure 9 exhibits the application prospect of this solar-driven MCH dehydrogenation system which can convert and store solar energy in practical operation.

### 4.3. Environmental Performance

**Figure 10:**
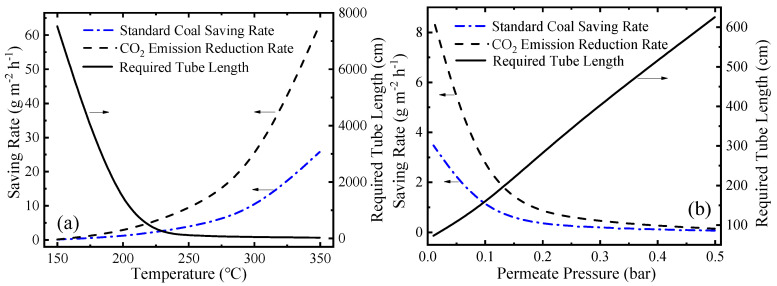
Variation of the standard coal saving rate (SCSR), the carbon dioxide emission reduction rate (CDRR), and the required tube lengths: (**a**) at different temperatures under 0.01 bar; and (**b**) under different permeate pressures at 250 °C.

In addition to thermodynamic performance, environmental performance is also significant for the evaluation of this system. This novel MCH dehydrogenation system is driven by solar energy so significant amounts of fossil fuel can be saved and a large amount of carbon dioxide emission can be reduced. The variation of SCSR (Equation (9)), CDRR (Equation (10)), and required tube lengths at different working conditions are shown in Figure 10. In Figure 10a, as the temperature goes up, the final conversion rate goes up, and the kinetic rate of reaction increases, so the required tube length decreases. In Figure 10b, lower permeate pressure means a higher conversion rate, a faster hydrogen separation rate and a faster reaction rate according to Equations (A1)–(A6) (given in Appendix A in detail), so the required tube length is shorter. As temperature increases or permeate pressure decreases, the conversion rate at per unit time and per unit mirror field area increases, which corresponds to more solar energy utilized. After assuming the energy consumed is provided by fossil fuel, the SCSR, and CDRR increase. The SCSR and CDRR can reach 25.73 and 63.03 g·m^−2^·h^−1^, respectively, at 350 °C 0.01 bar.

## 5. Conclusions

A novel MCH dehydrogenation system integrated with an HPM reactor driven by solar energy was proposed in this paper, which is to efficiently convert solar thermal energy into chemical energy for solar energy storage and hydrogen generation. Thermodynamic analysis of the system has been conducted based on numerical simulation. The highlighted conclusions are listed as follows:

(1) The conversion rate of MCH dehydrogenation with the assist of the HPM reactor can be improved, which can reach as high as 99.7% under 200 °C, 0.01 bar, compared to 8.65% in a traditional reactor without hydrogen separation at 200 °C.
(2) This novel system can efficiently utilize solar energy. The first-law of thermodynamic efficiency, solar-to-fuel efficiency, and exergy efficiency can reach 95.58%, 38.65%, and 94.22%, respectively. With a compressor to increase the partial pressure difference of hydrogen, the efficiencies can be improved due to the relatively high mechanical efficiency of the compressor compared with that of a vacuum pump.
(3) This is an environmentally friendly system, and it can save fossil fuels and reduce the emission of carbon dioxide. The SCSR and CDRR can be 25.73 and 63.03 g·m^−2^ ·h^−1^ at 350 °C 0.01 bar.

Based on the results of this study, this research provides further insights into efficient mid/low-temperature solar energy conversion and storage, which can also increase the energy level of solar energy to that of chemical energy. Therefore, this proposed system has great potential to be utilized as a promising approach for solar energy storage and high-purity hydrogen generation in the future.

## Nomenclature

*B*	dimensionless activation energy of adsorption for lumped equilibrium constant
*B* ^’^	dimensionless heat of adsorption for lumped equilibrium constant
*C*_p_	specific heat capacity (kJ/(mol K))
*d*_M_	thickness of membrane (m)
*E*	activation energy (J/mol)
*E*_x_	exergy (kJ/mol)
HHV	molar higher heating value (kJ/mol)
Δ*H*	enthalpy change (kJ/mol)
$\Delta h^{\prime }$	lumped heat of adsorption (kJ/mol)
*J*	hydrogen permeation flux (mol/m^2^/s)
*K*	equilibrium constant of the MCH dehydrogenation reaction
*K* ^’^	lumped equilibrium constant at reaction temperature
*K*_r_^’^	lumped equilibrium constant at reference temperature
*K*_A_	adsorption equilibrium constant of MCH (bar^−1^)
*K*_B_	adsorption equilibrium constant of TOL (bar^−1^)
*K*_C_	adsorption equilibrium constant of hydrogen (bar^−1^)
*k*_d_	apparent short-term deactivation constant (day^−1^)
*k*	rate constant at the reaction temperature (mol/g/s)
*k*_r_	rate constant at the reference temperature (mol/g/s)
*P*	pressure (bar)
*P*_0_	atmosphere pressure (bar)
*P*_r_	reaction pressure (bar)
*p*_A_	partial pressure of MCH (bar)
*p*_B_	partial pressure of TOL (bar)
*p*_C_	partial pressure of hydrogen (bar)
*Q*_preheat_	solar thermal energy input for raising the temperature of reactant (kJ)
*Q*_enthalpy_	solar thermal energy input for enthalpy change (kJ)
*Q*_sh_	thermal energy contained in gases after reaction (kJ)
R	universal gas constant (J/(mol K))
*R*_in_	inner radius (cm)
*R*_o_	outer radius (cm)
*r*	kinetic rate of the reaction (mol/g/s)
*T*_0_	room temperature (K)
*T*_H_	reaction temperature (K)
*T*_r_	reference temperature (K)
*T*_sun_	surface temperature of sun (K)
*t*_d_	online reaction deactivation time (day)
$W_{p,\mathrm{vacuum}}$	exergy consumed by vacuum pump to separate hydrogen (kJ)
$W_{p,\mathrm{compressor}}$	exergy consumed by compressor to feed MCH into reactor (kJ)
*q*_coal_	heating value of standard coal (kJ/kg)
Greek symbols	
*μ*	mass ratio of carbon dioxide emission from standard coal combustion (–)
*α*	conversion rate of methane (–)
*η*_c__→e_	conversion efficiency from standard coal to electricity (–)
*η*_c__→h_	conversion efficiency from standard coal to heat (–)
*η*_HHV_	First-law of thermodynamic efficiency with separation exergy (–)
*η*_HHV,real_	First-law of thermodynamic efficiency with real separation energy (–)
*η*_s__→f_	Solar-to-fuel efficiency with separation exergy (–)
*η*_s__→f,real_	Solar-to-fuel efficiency with real separation energy (–)
*η*_ex_	exergy efficiency (–)
*η*_abs_	absorption efficiency (–)
*η*_opt_	optical efficiency (–)
$\eta_{p_{1}}$	vacuum pump efficiency (–)
$\eta_{p_{2}}$	compressor mechanical efficiency (–)
*η*_s__→e_	Solar-to-electricity efficiency (–)
Superscript	
⊖	standard state
*	active sites of catalyst
Subscripts	
d	day
in	input, inside
init	initial
opt	optical
out	output, outside
p	vacuum pump/compressor
res	residual gas

## Appendix A

Kinetic model: According to the researches about MCH dehydrogenation reaction, the Langmuir–Hinshelwood–Hougen–Watson (LHHW) kinetic model fits well with the experimental kinetic data [40,41,42]. The kinetic reaction rate of MCH dehydrogenation reaction (Equation (1)) can be expressed as follows [40]:

(A1) $$r=-\frac{kK_{A}p_{A}(1-\frac{p_{B}p_{C}^{3}}{Kp_{A}})}{1+K_{A}p_{A}+K_{B}p_{B}+K^{\prime }p_{B}p_{C}^{2}}\left(1-k_{d}t_{d}\right)$$

(A2) $$k=k_{r}\exp\left[B\left(1-\frac{T_{r}}{T_{H}}\right)\right]$$

(A3) $$K=3600\exp\left[\frac{-217650}{R}\left(\frac{1}{T_{H}}-\frac{1}{650}\right)\right]$$

(A4) $$B=\frac{E}{RT_{r}}$$

(A5) $$K^{\prime }=K_{r}^{\prime }\exp\left[B^{\prime }(1-\frac{T_{r}}{T_{H}})\right]$$

(A6) $$B^{\prime }=\frac{\Delta h^{\prime }}{RT_{r}}$$

where *r* (mol g^−1^ s^−1^) is the reaction rate of MCH dehydrogenation; *k* is the rate constant (mol g^−1^ s^−1^); *K*_A_ (bar^−1^), *K*_B_ (bar^−1^), and *K*_C_ (bar^−1^) are the adsorption equilibrium constants for MCH, TOL, and hydrogen, respectively; *p*_A_, *p*_B_, and *p*_C_ are the partial pressures of MCH, TOL, and hydrogen, respectively; *K* is the equilibrium constant of the MCH dehydrogenation reaction; *K*′ and *K_r_*′ are the lumped equilibrium constants at reaction temperature and reference temperature, respectively; *k*_d_ (day^−1^) is the apparent short–term deactivation constant; *t*_d_ (day) is the online reaction deactivation time; *B* and *B*′ are the dimensionless activation energy and dimensionless heat of adsorption for lumped equilibrium constant, respectively; *T*_r_ (K) and *T*_H_ (K) are the reference temperature and reaction temperature, respectively; *k*_r_ is the rate constant at the reference temperature; *E* (J/mol) is the activation energy; *R* is the universal gas constant, taken as 8.314 J/(mol K); and $\Delta h^{\prime }$ is the lumped heat of adsorption. The values of the main parameters are listed in Table A1 [40]:

**Table A1:** The values of key parameters in the LHHW model.

Parameter	Value	Units
*k*_r_ × 10^5^	4.066 ± 0.44	mol g^−1^ s^−1^
*B*	7.65 ± 0.10	–
*K*_A_	40.9 ± 10.5	bar^−1^
*K*_B_	22.2 ± 7.05	bar^−1^
*K_r_^′^*	6.69 ± 1.43	bar^−3^
*B* ^′^	−24.0 ± 3.14	–
$\Delta h^{\prime }$	−123.4	kJ mol^−1^
*k*_d_	1.47 ± 0.17	day^−1^

Hydrogen separation model: According to the studies on Pd-based HPM, the hydrogen flux can be expressed as [43]:

(A7) $$J_{H_{2}}=\frac{k\left(P_{H_{2},\mathrm{in}}^{n}-P_{H_{2},\mathrm{out}}^{n}\right)}{d_{M}}$$

(A8) $$k=3.85\times 10^{-7}\exp\left(-\frac{18560}{8.314\times T_{H}}\right)$$

where $J_{H_{2}}$ (mol H_2_ m^−2^ s^−1^) is the hydrogen flux of the Pd-Ag membrane; *n* is an exponent, taken as 0.5; $P_{H_{2},\mathrm{in}}$ and $P_{H_{2},\mathrm{out}}$ are the hydrogen partial pressures (Pa) on the feed side and the permeate side of HPM reactor, respectively; and *d*_M_ (m) is the thickness of HPM.

*Thermodynamic model:* In Equations (2) and (4)‒(6), *Q*_preheat_ is the solar thermal energy consumed for raising the MCH temperature from room temperature to the reaction temperature and it can be expressed as:

(A9) $$Q_{\mathrm{preheat}}=n_{C_{7}H_{14},\mathrm{init}}\cdot \int_{T_{0}}^{T_{H}}C_{p,C_{7}H_{14}}dT$$

where $n_{C_{7}H_{14},\mathrm{init}}$ is the initial molar amount of MCH; *T*_0_ is the room temperature, taken as 25 °C; and $C_{{p,C}_{7}H_{14}}$ is the specific heat capacity of MCH. *Q*_enthalpy_ is the solar thermal energy consumed for the enthalpy change of MCH dehydrogenation, which can be expressed as:

(A10) $$Q_{\mathrm{enthalpy}}=\Delta H\cdot n_{C_{7}H_{14},\mathrm{init}}\cdot \alpha$$

where ΔH and α are the enthalpy change and conversion rate of MCH dehydrogenation, respectively. *W*_p,vacuum_ is the exergy consumed by a vacuum pump for hydrogen separation, which can be expressed as:

(A11) $$W_{p,\mathrm{vacuum}}=n_{H_{2},\mathrm{out}}\cdot RT_{0}\ln\left(P_{0}/P_{H_{2},\mathrm{out}}\right)$$

where $n_{H_{2},\mathrm{out}}$ is the molar amount of hydrogen separated; *P*_0_ is the atmospheric pressure; and $P_{H_{2},\mathrm{out}}$ is the hydrogen permeate pressure. *W*_p,compressor_ is the exergy consumed by a compressor for feeding the MCH into the reactor, which can be expressed as:

(A12) $$W_{p,\mathrm{compressor}}=n_{C_{7}H_{14},\mathrm{init}}\cdot RT_{0}\ln\left(P_{r}/P_{0}\right)$$

where *P*_r_ is the feeding pressure. In Equation (7), *Q*_wh_ is the thermal energy contained in the products after the reaction, expressed as:

(A13) $$Q_{\mathrm{wh}}=n_{H_{2}}\int_{T_{0}}^{T_{H}}C_{{p,H}_{2}\left(g\right)}dT+n_{C_{7}H_{8}}\int_{T_{0}}^{T_{H}}C_{p,C_{7}H_{8}\left(g\right)}dT+n_{C_{7}H_{14}}{}_{,\mathrm{res}}\int_{T_{0}}^{T_{H}}C_{{p,C}_{7}H_{14}\left(g\right)}dT$$

where $C_{{p,H}_{2}\left(g\right)}$, and $C_{p,C_{7}H_{8}\left(g\right)}$ are the specific heat capacities of hydrogen and TOL, respectively.
